# Spatiotemporal patterns of extreme sea levels along the western North-Atlantic coasts

**DOI:** 10.1038/s41598-019-40157-w

**Published:** 2019-03-04

**Authors:** Sanne Muis, Ning Lin, Martin Verlaan, Hessel C. Winsemius, Philip J. Ward, Jeroen C. J. H. Aerts

**Affiliations:** 1 Institute for Environmental Studies (IVM), Vrije Universiteit Amsterdam, Amsterdam, The Netherlands; 2 Department of Civil and Environmental Engineering, Princeton University, Princeton, New Jersey USA; 3 Deltares, Delft, The Netherlands; 4 TU Delft, Delft, The Netherlands

## Abstract

The western North-Atlantic coast experienced major coastal floods in recent years. Coastal floods are primarily composed of tides and storm surges due to tropical (TCs) and extra-tropical cyclones (ETCs). We present a reanalysis from 1988 to 2015 of extreme sea levels that explicitly include TCs for the western North-Atlantic coastline. Validation shows a good agreement between modeled and observed sea levels and demonstrates that the framework can capture large-scale variability in extreme sea levels. We apply the 28-year reanalysis to analyze spatiotemporal patterns. Along the US Atlantic coasts the contribution of tides can be significant, with the average contribution of tides during the 10 largest events up to 55% in some locations, whereas along the Mexican Southern Gulf coast, the average contribution of tides over the largest 10 events is generally below 25%. At the US Atlantic coast, ETCs are responsible for 8.5 out of the 10 largest extreme events, whereas at the Gulf Coast and Caribbean TCs dominate. During the TC season more TC-driven events exceed a 10-year return period. During winter, there is a peak in ETC-driven events. Future research directions include coupling the framework with synthetic tropical cyclone tracks and extension to the global scale.

## Introduction

The western North-Atlantic coasts (including the Caribbean Sea and the Gulf of Mexico) have experienced major coastal flooding, primarily driven by tropical cyclones (TC)^1^. When Hurricane Katrina struck New Orleans in 2005, more than a million people in the region were displaced and estimated damages exceed $100 billion^2^. Hurricane Sandy hit the New York region in 2012 and caused $50 billion in economic losses^3^. With estimated damages exceeding $300 billion, 2017 is the costliest season on the Atlantic record^4,5^. In 2018 Hurricanes Florence and Michael both brought life-threatening storm surge. At mid-latitudes, extra-tropical cyclones (ETC) can also induce major flooding^6^. While ETCs generally cause lower surge heights than TCs, the former are more frequent, have longer durations, and impact much larger areas^7^. In addition, astronomical tide can significantly affect the flood level; the flooding associated with Hurricane Sandy was aggravated due to its coincidence with spring high tides^8^. Therefore, it is essential to include all these drivers when analyzing the variability in extreme sea levels.

Mapping the spatiotemporal drivers of sea level extremes is important for risk management and can contribute to understanding spatial variability and developing best practices for local modelling. Previous studies from local to global have used observations to analyze the drivers of extremes^9–12^. Using observations for analyzing extremes can be problematic, especially in the case of TCs. As the largest effects of TCs are within a few tens of kilometers of landfall^13^, the spatial coverage of tide gauge networks is scarce, and tide gauges often fail during extreme weather conditions, TC surges are generally recorded at a very limited number of tide gauges^14,15^. Hydrodynamic modelling has therefore become a valuable approach. Applications of hydrodynamic models have traditionally been limited to the local-scale^16–22^, but recently studies have applied such models at continental to global scale^23–28^. However, many of the large-scale studies have either focused specifically on TCs^29^ or have been based on relatively coarse climate reanalysis data^25^. As climate reanalysis datasets, such as ERA-Interim^30^, poorly represent the intensities of TCs^31^, large scale analyses of extreme sea levels that are based on them tend to poorly represent storm surge from TCs. Zhang and Sheng^32^ presented the spatial distribution of return periods of extreme sea levels induced by TCs and ETCs over the eastern continental shelf of North America. However, this study has not analyzed the drivers of extreme sea levels and their spatiotemporal patterns in detail.

In this letter we study the spatiotemporal patterns of sea level extremes along the western North-Atlantic coasts based on a hydrodynamic modeling approach. We develop a complete reanalysis of extreme sea levels along the western North-Atlantic coasts for the period 1988–2015, including tides, and surges from TCs and ETCs. To achieve this, we improved upon the global framework of Muis *et al*.^25^ by explicitly modeling TC surges with high-resolution wind and pressure fields based on a parametric model. To assess the applicability of the reanalysis dataset, we validate the modeled sea levels against observations. Subsequently, we study the spatiotemporal patterns and drivers of extremes using the validated dataset with surge and total sea levels. Note that the contribution of waves to extreme sea levels is ignored, although in some places waves are important drivers of extremes.

## Methods

### General approach

**Figure 1:**
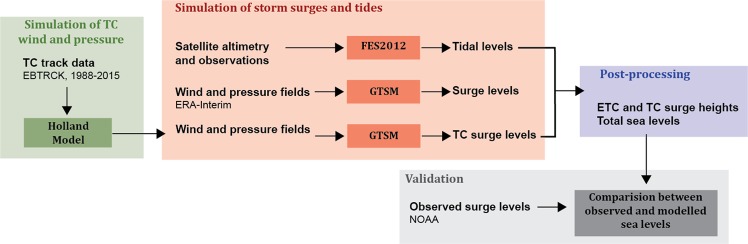
Flowchart of the model framework. TC track data are taken from the Extended Best Track Dataset (EBTRCK).

For this application, we modified the global framework of Muis *et al*.^25^ for better representation of TCs (Fig. 1) and used it to simulate surge levels for the period 1988–2015. The core of this framework is the Global Tide and Surge Model (GTSM)^33^. The approach is designed for effective large-scale analysis, and tides, storm surges, and TC storm surges are modelled separately. Therefore, we exclude non-linear interaction between tide and surge and the effect of wave setup, which may be important in some regions^29^.

### Modelling of storm surges and extreme sea levels

Storm surges are simulated with the Global Tide and Surge Model (GTSM)^33^ based on the Delft3D Flexible Mesh software (Delft3D FM)^34^. We focus on the spatial domain within 5°N and 50°N and 100°E and 50°E. To simulate TC storm surges, GTSM is forced with EBTRCK wind and pressure fields based on the Extended Best Track Dataset (EBTRCK)^35^. For the period 1988–2015, EBTRCK contains 377 Atlantic TCs. We select the 219 TCs that come within 100 km of the land. For each track, we use 30 hours before the track comes within 100 km of land until the end of the track. The 6-hourly coordinates, 1-minute maximum wind speed, pressure drop, and maximum wind radius for each storm are interpolated to a 1 hourly time interval assuming a constant translation speed and using cubic spline interpolation. Given these storm characteristics, we apply a parametric TC model(i.e. the Holland model)^36^ to estimate the gradient wind and sea-level pressure fields for each TC. The wind speed at the gradient level is converted to the surface level (at 10 m) using a wind speed reduction factor of 0.85^37^. The asymmetry in the surface wind is accounted for by adding estimated surface background winds to the wind field^38^. We compute wind and pressure fields, that are input to GTSM, using a polar co-ordinate grid centered on the TC eye (so-called spiderweb grid)^39^ with a radial resolution of 1 km and a tangential resolution of 5°. The 1-minute wind is converted to a 10-minute average by a reduction factor of 0.915^40^. In Delft3D FM we define the translation from wind speed into wind stress based on the formulation of Garrat^41^ with a cap of 0.0025 on the drag coefficient^38^, which occurs when the wind speed is greater than 25 m/s. We use this formulation rather than the frequently applied Charnock formulation^42^ because Powell *et al*.^43^ suggested that the drag coefficient levels off at a high wind speed.

In addition, we simulate storm surges using wind and pressure fields from ERA-Interim climate reanalysis data^30^ developed by the European Centre For Medium-Range Weather Forecasts (ECMWF). ERA-Interim has a temporal resolution of 3 hours and a spatial resolution of 0.75 × 0.75°. For consistency with ECMWF’s climate model, we apply the Charnock^42^ drag formulation with a Charnock parameter of 0.041. This stet-up is comparable with Muis *et al*.^25^, but for this application we store the output data for all the coastal cells of the GTSM grid (every ~5 km along the coast).

We combine the EBTRCK and ERA-Interim simulations by taking the highest of the two surge heights for each time step. Subsequently, the total sea level is calculated by superimposing the surges with astronomical tides. Tides are simulated based on the FES2012 model^44^. FES2012 is a global tidal model that assimilates satellite altimeter data and has a gridded resolution of 1/16°. We use mean sea level, as defined by the GEBCO bathymetry^45^, as vertical datum.

### Validation of the model framework

An important step in our analysis is the validation of our model framework. This is done by evaluating the modelled sea levels against literature and observations. Time-series of predicted tides and observed sea levels are obtained from NOAA tide gauges. The validation consists of two steps: first, we analyze four major historical TC events in detail, and subsequently we validate all TC events.

We select four major historical TC events to evaluate the EBTRCK simulations: Hurricane Katrina (2005) and Hurricane Ike (2008) at the Gulf Coast; and Hurricane Irene (2011) and Superstorm Sandy (2012) at the Mid-Atlantic coast. We validate the winds from EBTRCK and ERA-Interim against the H*wind dataset^46^. The H*wind dataset is based on various surface wind observations and has been applied for wind analysis and surge simulations^47,48^. All wind data are linearly interpolated to the H*wind grid for validation. Surge heights are validated against literature and tide gauge observations.

In addition, we validate all the simulated TC surges and total sea levels. To validate the TC surges, we select the tide gauges that are located within 500 km of the TC track. To validate the total sea level, we apply a peaks-over-threshold (POT) method to the observed time-series. For each location, we set the threshold at the level to match an average of 3 events per year^49^. Consistent with previous studies, time-series are declustered using a 3-day window to ensure independent events^28,50^. Next, we extract the corresponding modelled sea levels for each observed sea level during the event.

Time-series of predicted tides and observed sea levels are obtained from NOAA tide gauges (https://tidesandcurrents.noaa.gov). Using a simplified method we calculate skew surge by subtracting the daily maxima of predicted tides from the daily maxima of observed sea levels, as it is a more robust measure than the tidal residual^51^. All sea levels are referenced above mean sea level; therefore we remove the linear trend in monthly mean sea level. Using a 12-month running mean, we also remove the annual variations in mean sea level. Low-frequency fluctuations in mean sea level are largely caused by variations in ocean temperatures, salinities, and currents^52,53^ which are processes that are not included in GTSM.

We use various indicators for validation. For the wind simulations, we calculate Root Mean Squared Error normalized over the maximum wind speed in H*wind (NRMSE). We also calculate Pearson’s correlation coefficient for wind intensities above 10 m/s only. For the surge and sea level simulations, we calculate the mean bias [m], the mean absolute error [m], and Pearson’s correlation coefficient over the observed and modelled maxima. We also show the hit rate [%], which indicates the percentage of tide gauges where modelled maxima fall within the 25% error margins of the observed maxima.

### Analysis of spatiotemporal drivers of extremes

**Figure 2:**
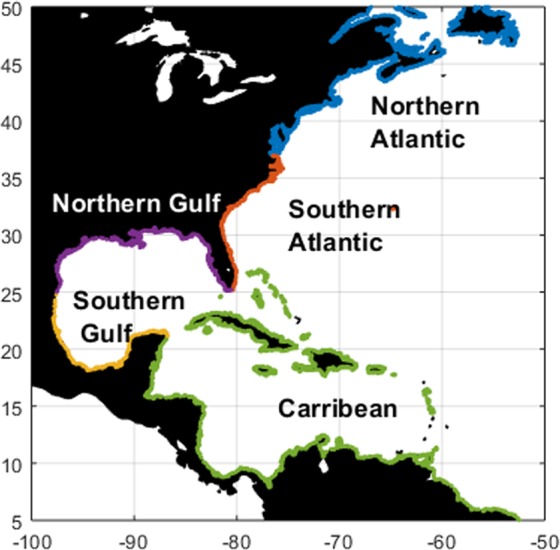
Maps showing how the different sub-basins are defined.

After validating the modelled sea levels, we study the spatiotemporal drivers of sea level extremes along the western North-Atlantic coasts. To this end, we define five sub basins: Northern Atlantic (US), Southern Atlantic (US), Northern Gulf of Mexico (US), Southern Gulf of Mexico (Mexico), and the Caribbean (Fig. 2). The analysis is based on a peaks-over-threshold (POT) approach^49^ and consists of the following steps:

1. Selection of extreme events. To select the extreme events, we apply a peaks-over-threshold (POT) approach^49^ to find the peaks that exceed the 99th percentile^54^. To ensure independent events, we use a decluster time of 3 days between events^28,50^. The analysis is applied to both storm surge levels and total sea levels at each locations.
2. Estimate return periods of extreme events. For each location, we fit a Generalized Pareto Distribution (GPD) to the events derived in step 1, and calculate the 1-year, 2-year and 10-year return level from the fitted distribution. Subsequently, we assess for each event at each locations the corresponding return period. Given the limited length of our simulation, we do not focus on the probabilities of events that exceed the 10-year return level.
3. Analyze drivers of extreme events. At each location we select the 10 largest extreme events (with  the corresponding return periods) and examine the average contribution of each driver over those events (i.e. tides, ETCs and TCs). In addition to the validation explained in 2.3, we computed at each location how the GDP fit changes when explicitly including TCs in the simulations with respect to simulations that are based on ERA-Interim. This to test how sensitive the results are to different forcing, which can provide insights at which return level TCs play an important role for different locations.
4. Assess temporal variability of extreme events. We also assess the seasonality of the occurrence of extreme events by counting the number of events driven by either TCs or ETCs for different return periods in each season for the different regions.

## Results and Discussion

### Validation of the extreme sea levels

#### Validation of major TC events

**Figure 3:**
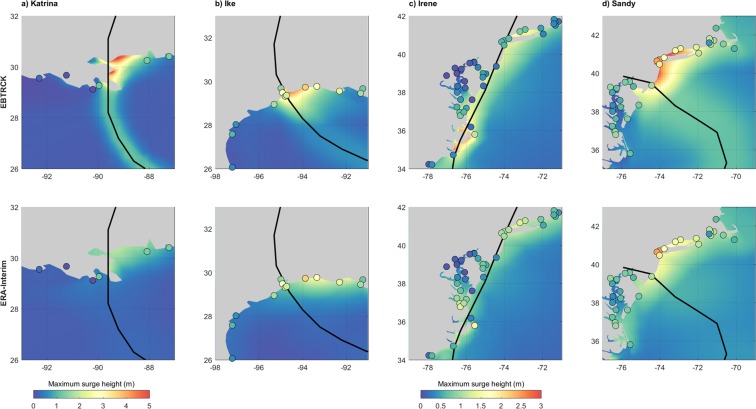
Validation of the maximum surge heights. The upper and lower panels show the maximum surge heights based on EBTRCK and ERA-Interim for respectively (**a**) Hurricane Katrina, (**b**) Hurricane Ike, (**c**) Hurricane Irene, and (**d**) Hurricane Sandy. The colored dots shown the highest observed surge heights at tide gauge stations.

Hurricane Katrina: Hurricane Katrina made landfall near New Orleans on 29 August 2005 and caused devastating losses throughout south-eastern Louisiana and Mississippi^2^. The correlation between the H*wind and EBTRCK is 0.67, compared to 0.17 for ERA-Interim (Table 1). EBTRCK performs better than ERA-Interim, but overestimates wind intensities on the left side of the TC eye, while underestimating wind intensities on the right side (Figs S1a and S2a). This is a limitation of parametric TC models in general, which are based on an idealized representation of TCs with a well-defined and symmetrical structure^55^. Inaccuracies may also derive from the simple approximation of the background winds^38^. Forcing GTSM with EBTRCK results in a maximum surge of 5.5 m, compared to 2.2 m for ERA-Interim. The highest modelled surges occurred in the areas near Breton Sound, Chandeleur Sound, and St. Louis Bay (Fig. 3a). There are 10 tide gauge stations available within 500 km. At those locations, the EBTRCK modelled surge heights have a mean bias of 0.03 m at the peak of the event (Table 1). The performance however varies across locations (Fig. S3). Time-series of modelled and observed surge levels shows that while the performance in terms of peak surge is relatively good, the onset of the timing can be off and the onset of the surge is generally too low (Fig. S4). Based on a detailed regional hydrodynamic model, Dietrich *et al*.^56^ reported a maximum surge up to 8.8 m in low-lying bays and 6.0 m along the shelf of the Mississippi-Alabama coast. While our results do not capture such extreme surge heights in semi-enclosed and low-lying bays, they do provide a good approximation of the surge heights along the open coasts.

**Table 1:** Performance of the wind model and surge model for four major TCs. S.D. indicates the standard deviation across the tide gauge stations.

*Wind simulations*		Katrina	Ike	Irene	Sandy
EBTRCK	NRMSE (%)	17.2	15.1	28.6	22.7
	Pearson corr. (r)	0.67	0.67	0.38	0.31
ERA-Interim	NRMSE (%)	21.6	23.0	30.2	21.8
	Pearson corr. (r)	0.18	0.06	0.26	0.03
***Surge simulations***					
EBTRCK	Mean bias (m)	−0.03 S.D. 0.17	−0.13 S.D. 0.67	0.20 S.D. 0.24	0.00 S.D. 0.41
	Mean absolute error (m)	0.13 S.D. 0.11	0.55 S.D. 0.37	0.24 S.D. 0.20	0.32 S.D. 0.25
	Hit rate (%)	60	31	35	42
	Pearson corr. (r)	0.95	0.84	0.83	0.81
ERA-Interim	Mean bias (m)	0.03 S.D. 0.34	−0.49 S.D. 0.62	0.07 S.D. 0.25	−0.08 S.D. 0.34
	Mean absolute error (m)	0.24 S.D. 0.23	0.61 S.D. 0.49	0.20 S.D. 0.17	0.27 S.D. 0.21
	Hit rate (%)	40	31	47	44
	Pearson corr. (r)	0.77	0.73	0.79	0.75
	No. of tide gauges within 500 km radius of TC track	10	13	46	45

Hurricane Ike: On 11 September 2008, Hurricane Ike made landfall near Galveston and caused extensive damage along the coasts of Texas and Louisiana^57^. The improvement in the representation of the wind field when comparing ERA-Interim and EBTRCK to H*wind is similar to that for Hurricane Katrina, with an increase in correlation from 0.06 to 0.67 (Table 1). Ike’s central wind field is well represented by EBTRCK, although the parametric model has difficulties with predicting winds further away from the TC eye, as well as with capturing the asymmetry (Figs S1b and S2b). The highest observed surge was 5.3 m^58^. Forcing GTSM with EBTRCK results in a maximum surge of 4.8 m just north of Galveston Bay (Fig. 3b), compared to 2.6 m for ERA-Interim. In comparison with observations from 13 tide gauge stations, EBTRCK performs better than ERA-Interim (Table 1), although the performance varies strongly (Fig. S5). A reason for the strong variability in performance is the complex physics of Ike’s surge. The highest surges occurred in semi-enclosed bays and many areas experienced a forerunner surge hours before landfall^47,59^. This is not well captured by GTSM and neither is the timing of the peak of the surge (Fig. S6). Yet, the spatial pattern of the maximum surge heights is in agreement with observations, as well as the more detailed hydrodynamic model from Hope *et al*.^47^.

Hurricane Irene: Hurricane Irene made landfall in North Carolina and moved north-northeastward along the Mid-Atlantic coast, transitioning to an ETC early on 29 August 2011^60^. Due to this transitioning, EBTRCK does not fully represent Irene’s wind field (Figs S1c and S2c). Although the increase in performance from ERA-Interim to EBTRCK is smaller than as for Katrina and Ike (Fig. S2c), EBTRCK has a better agreement with H*wind with a correlation of 0.38 compared to 0.26 for ERA-Interim (Table 1). The maximum surge occurred at the North-Carolina coast and had a magnitude of 2.8 m and 1.5 m for EBTRCK and ERA-Interim, respectively (Fig. 3c). The EBTRCK maxima are in better agreement with observed maxima that go up to 3.4 m^60^. Due to the limited number of observations in the most impacted region, no large improvement in performance is found when comparing EBTRCK to ERA-Interim. However, the spatial pattern in the EBTRCK simulation is in line with the detailed hydrodynamic model results from Orton *et al*.^61^. Time-series of observed and modeled surge levels also compare relatively well, although at one particular tide gauge (located near the Oregon Inlet) the timing of the peak is off (Figs S7 and S8).

Hurricane Sandy: In October 2012, Hurricane Sandy hit the coasts of New Jersey and New York. EBTRCK’s performance is considerably better than that of ERA-Interim (Table 1). Similar to Irene, Sandy was transitioning into an ETC when making landfall and did not have the typical structure of a TC (Fig. S1d). Compared to H*wind, EBTRCK overestimates the wind intensities (Fig. S2d). The highest surge occurred along the coast of New Jersey, New York and Connecticut (Fig. 1d). Observed surge heights are 2.9 m at The Battery (NY), 2.8 m at New Haven (NY), and 1.7 m at Atlantic City (NJ), comparable to the EBTRCK surge heights of 2.7 m, 2.5 m, and 2.0 m for these locations respectively. With surge heights of 1.7 m, 1.6 m, and 1.3 m respectively, ERA-Interim results in a larger underestimation. Time-series show an accurate timing of the peak, although the peak is generally too abrupt (Fig. S9). Generally, surge heights are underestimated in shallow and topographically more complex areas that require a higher resolution (Fig. S10).

#### Validation of the surge and total sea levels

**Figure 4:**
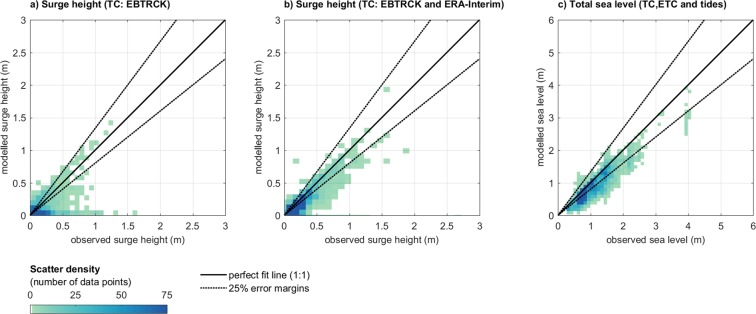
Scatter density plots of modeled and observed maxima for: (**a**) TC surge heights based on EBTRCK; (**b**) TC surge based on EBTRCK and ERA-Interim combined; and (**c**) extreme total sea levels derived from applying POT to the combined surge and tides time series (EBTRCK, ERA-Interim and FES2012). Colors express the data density using 0.10 m × 0.10 m bins for panels a and b, and 0.15 m × 0.15 m bins for panels c. The solid black line depicts the perfect fit, while the dashed black line depicts the 25% error margins.

As a next step, we validate all EBTRCK surge heights using over 2000 observations (122 tide gauge stations; 158 TCs) in the Caribbean and East Coast regions. Averaged across all observations, there is a reasonable agreement with a bias of 0.15 m and a correlation of 0.53 (Table S1). The scatter density plot in Fig. 4a shows a good agreement for maxima above 0.5 m, although some of the observations are not reproduced well. If we exclude the stations located behind barrier islands or in semi-enclosed bays, areas which are not well-resolved by GTSM, the correlation increases to 0.74. Furthermore, EBTRCK stops recording as soon as a storm is no longer classified as a TC. Many TCs weaken rapidly when making landfall and the track data of some TCs ends before the occurrence of the peak surge. Moreover, the parametric model has high accuracy in reproducing the wind near the TC eye, but errors increase with distance^62^. In contrast, ERA-Interim poorly reproduces the winds near the TC eye but performs better further away (Fig. S2). By combining the EBTRCK and ERA-Interim simulations (taking the highest surge at each time step), the model performance improves considerably (Fig. 4b). This is indicated by an increase in the correlation from 0.53 to 0.77 (Table S1). The performance particularly improves further than 250 km away of the TC track and for stations not located behind barrier island and in semi-enclosed areas, indicated by correlation coefficients of respectively 0.81 and 0.88 (Table S1).

We also validate the total sea levels including TCs, ETCs and tides using over 4000 observations from 220 tide gauge stations. Figure 4c shows that the modeled sea levels are in line with the observations. Averaged across all observations, there is a bias of 0.22 m and a correlation of 0.89 (Table S1). There is no major difference in performance for the different sub-basins with correlations varying between 0.7 and 0.8 (Fig. S11).

#### Sensitivity of return periods

**Figure 5:**
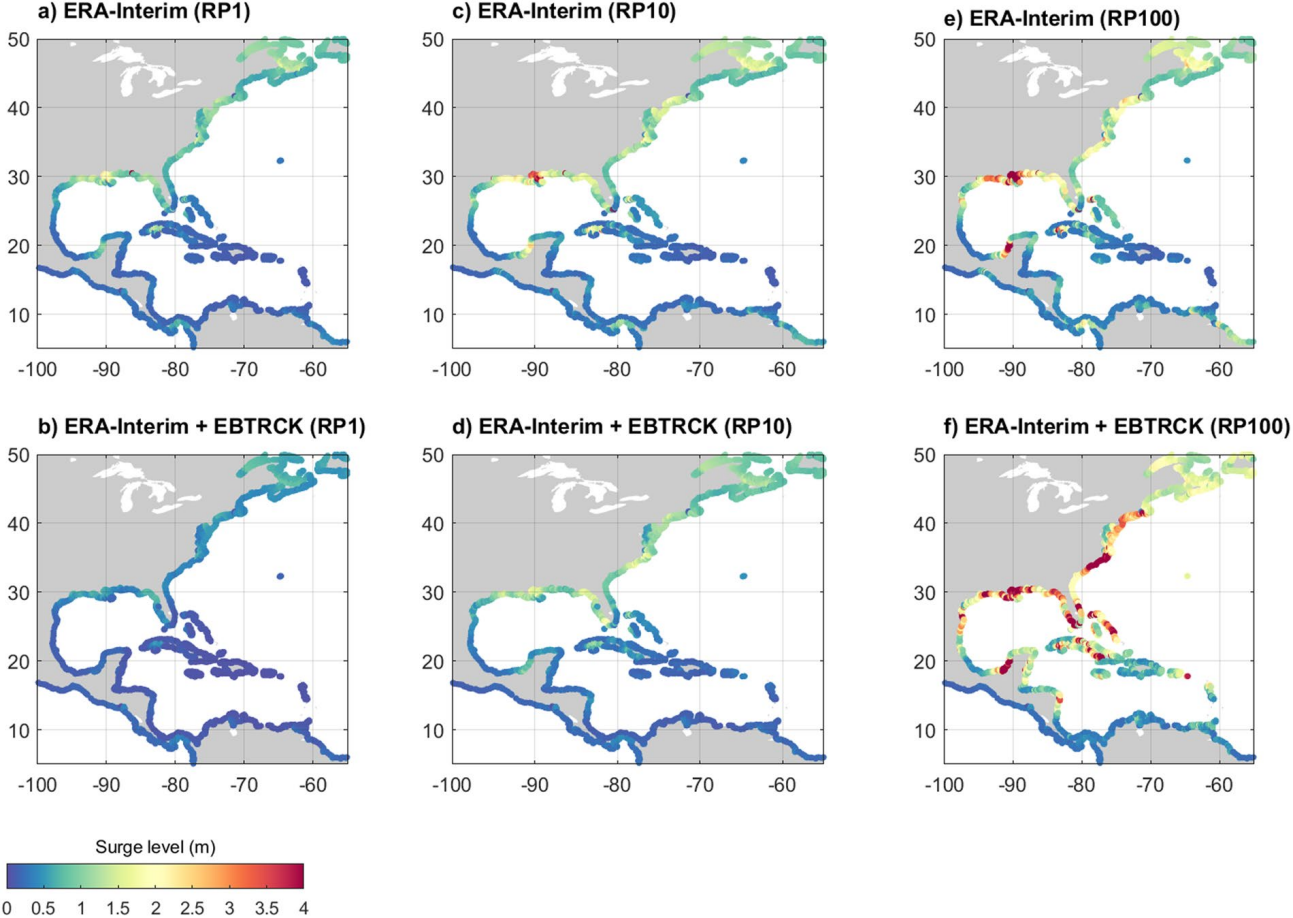
Sensitivity of the surge heights for the 1-year, 10-year and 100-year return period for explicitly including TCs. The upper panels (a,c,e) show the return periods for the GDP fit based on the surge peaks derived from ERA-Interim, whereas the bottom panels (b,d,f) show the return periods for the GDP fit based on the surge peaks derived from ERA-Interim and EBTRCK combined.

Finally, we assess how sensitive the estimation of return period is to a better representation of TCs. Figure 5 shows how surge levels for the 1-year, 10-year and 100-year return period change when combining the ERA-Interim simulations with the EBTRCK simulations. It shows that while surge levels for the smaller return periods do not changes much, explicitly including TCs can result in an increase of the 100-year surge levels exceeding 1 m. There is major impact at the coast of the south of the US (i.e. Texas, Louisiana, Florida), but also along the coast of the Mid-Atlantic (i.e. North Carolina, Virginia). Also in parts of the Mexican and Caribbean coastline there is a relatively large change. Figure S12 shows at which return period the GDP fit based on the surge peaks derived from EBTRCK and ERA-Interim combined exceed the GDP fit based on the surge peaks derived from ERA-Interim. We note that the dataset does not include information to reliably estimate the return periods of the most extreme events due to its relatively short length of the simulations^63^. As such, this analysis mainly indicates regions that were struck by a major TC in the period 1988–2015 and those regions that were not. It does however indicate that even for this relatively short length there is a strong increase in the surge return levels when explicitly including tropical cyclones.

### Understanding sea level extremes

#### Drivers of extreme events

**Figure 6:**
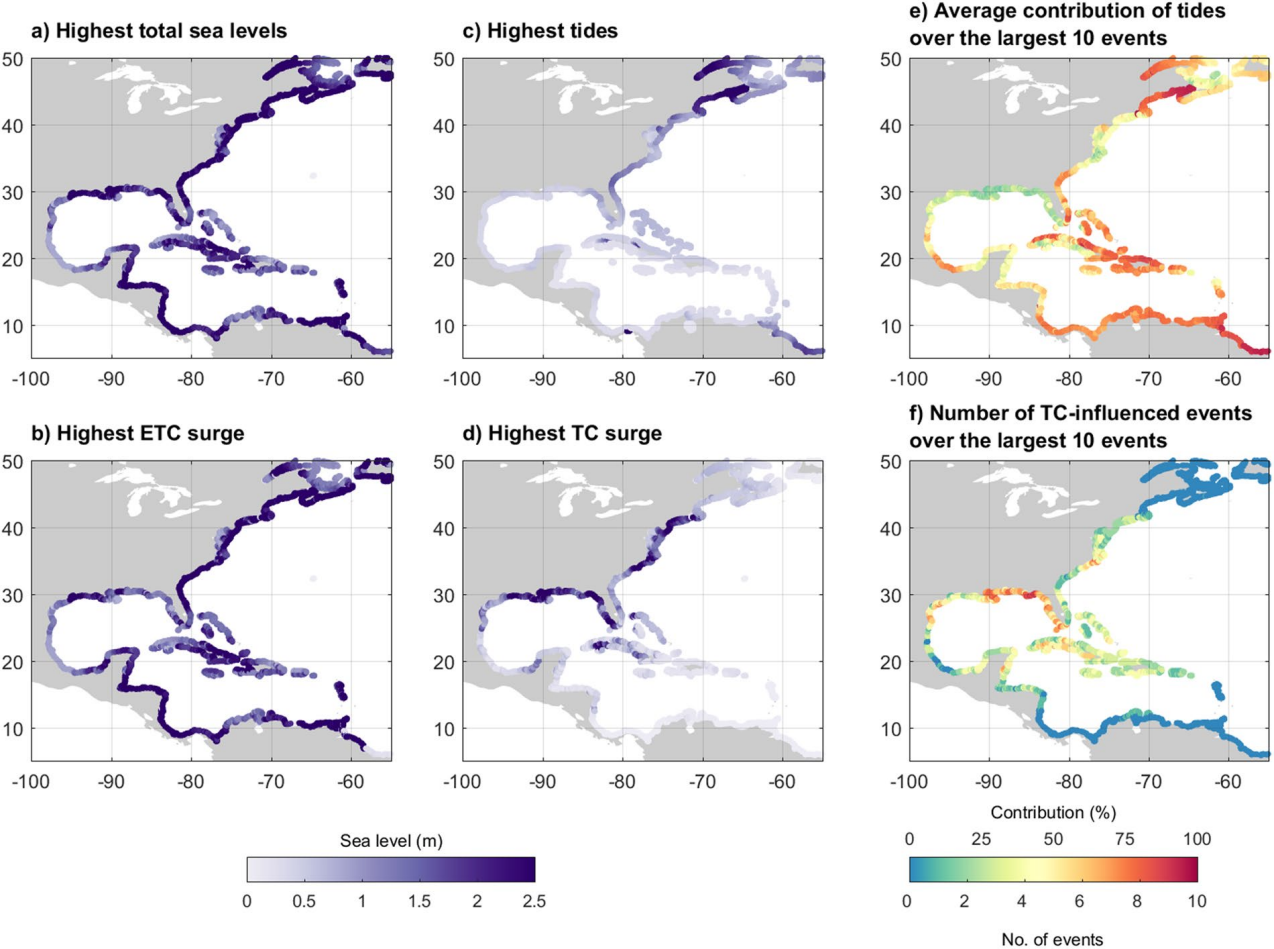
Maxima over the period 1988–2015 for: (**a**) total sea levels and its components; (**b**) ETC surges; (**c**) tides; and (**d**) TC surges. All surges within 500 km of the TC tracks are considered to be driven by TCs. To assess the drivers of the most extreme events, (**e**) shows the average contribution of tides over the 10 largest events, whereas (**f**) shows the number of TC-influenced events over the largest 10 events.

As a first step of analyzing the spatial patterns of the drivers of extreme sea levels, we map the highest total sea level and its drivers (i.e. tides, ETC and TC surges) over the period 1988–2015 (Fig. 6a–d; see Fig. 2 for the definitions of the sub-basins). Results show that along the US Northern Atlantic coastline the highest total sea levels exceed 2 m as a result of a large tidal range. Along the coast of the Mexican Southern Gulf and the Caribbean, total water levels are lower. This is linked with a relatively small tidal range in those areas with the highest tides generally below 1 m. Some parts of the US Northern Atlantic that are located north of New Jersey experience tides over 3 m. The highest storm surges induced by ETCs have a range of 1–2 m. Our results show large ETC effects can go as far south as 25°N. This may be linked with the occurrence of cold surges, which are a dominant feature of mid-latitude tropical interaction and affect the Gulf of Mexico during winter^64,65^. Along the Caribbean coasts, surges are generally low. This is because the Caribbean coastline is characterized by steep slopes, whereas the Gulf and Atlantic coastlines are characterized by shallow bathymetry and wide continental shelf. Surges induced by TCs are typically higher and can go up well beyond 2 m. The northern Gulf Coast and the Northern-Atlantic Coast, e.g. from Texas to North Carolina, in particular have experienced high TC surges up to 5 m.

Various patterns emerge when analyzing the average contribution of each driver over the largest 10 events (Fig. 6e,f; see Fig. 2 for the definition of sub-basins). Especially along the US Northern Atlantic coastlines, extremes are largely driven by tides: averaged over the largest 10 events, the relative contribution of tides to total sea levels in this region is on average 55% and 45% in respectively the US Northern Atlantic and US Southern Atlantic, but exceeds 80% in some parts of these sub basins. Along the Southern Gulf coast, the average contribution of tides over the largest 10 events is generally below 25%. At mid-latitudes, extremes are most likely caused by ETCs which count for on average 8.5 of the 10 largest events in the US Northern Atlantic (>37°N). In the US Southern Atlantic this is 6.0 events on average. For the Mexican Southern Gulf Coast, TCs are the most important driver of extreme events with an average of 6.2 TC-influenced events out of the largest 10 events. The high frequency of ETCs and the relatively short period considered leads to a relatively high importance of ETCs. When analyzing more extreme events, TCs become a more important driver. On average, 1 out of the 3 largest events is driven by TCs. In the Mexican Southern Gulf, on average 2.2 out of the 3 largest events is driven by TCs, compared to 6.2 out of the 10 largest events. The average contribution of tides is smaller for the largest 3 events than for the largest 10 events with contributions of respectively 36% and 55%. This indicates that the largest events are more strongly driven by storm surges, especially TC surges.

#### Temporal variability of extreme events

**Figure 7:**
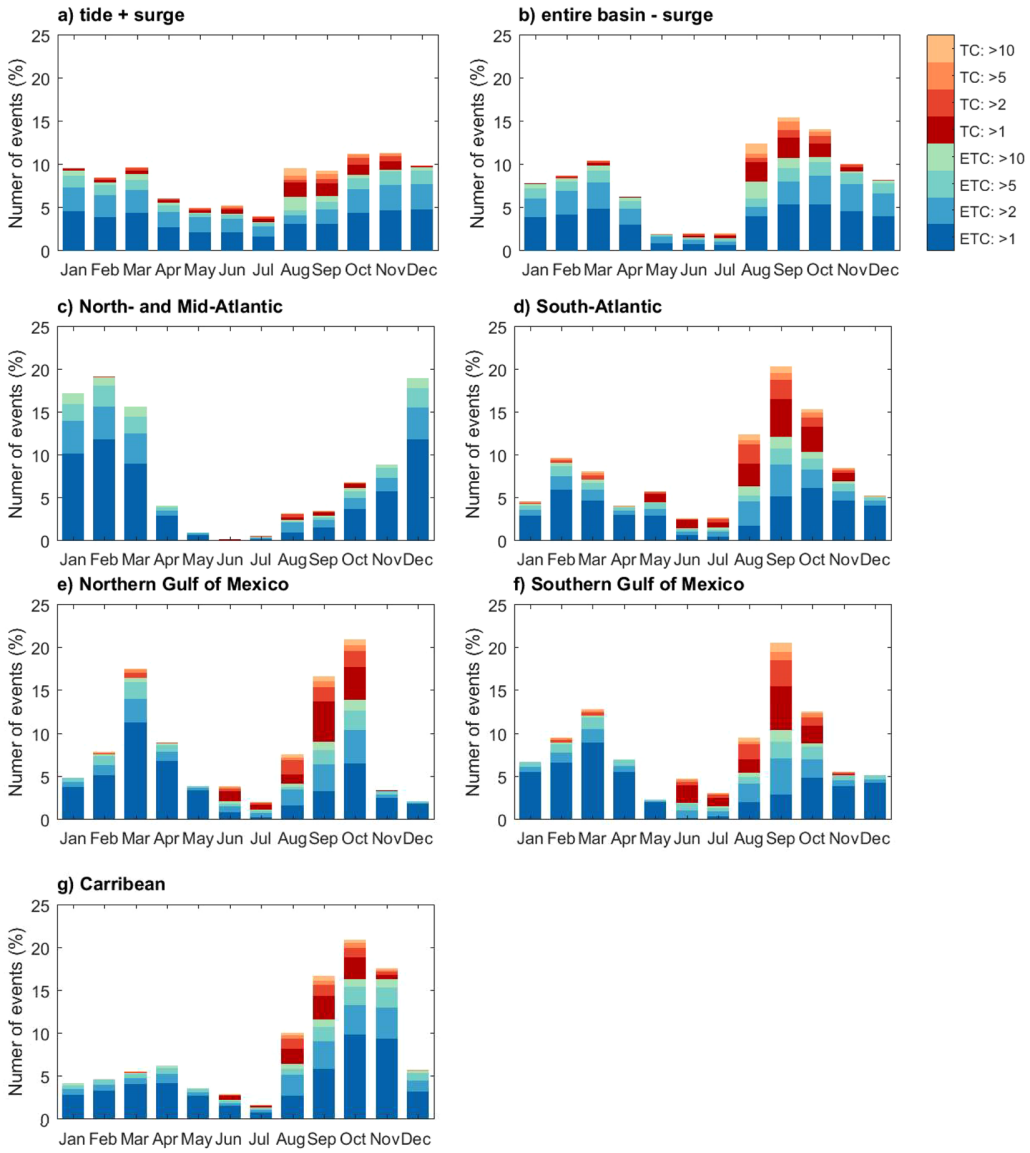
Number of extreme events per month. Panels a and b shows the number of sea level and surge level events for the entire basin, whereas the other panels show the number of surge event for the different sub-basins. Figure 2 defines the sub-basins.

The temporal variability of the set of events is analyzed by counting the numbers of events associated with specific return periods for each month. The result indicates a clear seasonality with fewer events in spring and early summer, while in late summer to early fall and in winter there are more events. Moreover, the levels of the events in late summer to early fall and winter correspond to higher return periods (Fig. 7a). For example, in May 2% of the events for total water levels (i.e., tide and surge) in that month have a return period higher than 10 years, while in August 26% of the events in that month have a return period higher than 10 years. The seasonal patterns become more distinct when analyzing the surge component only (Fig. 7b). It also shows that during the Atlantic TC season, which peaks from August to September, there is a larger fraction of TC-driven events with return periods that exceed 10 years. Figure 6c–g shows that there are distinct seasonal patterns between the different sub-basins. In the US Northern -Atlantic, there is a concentration of events in winter, while almost no events occur from April to July. In the Caribbean, the events concentrate in late summer, which suggests they are largely driven by TCs. Along the Gulf of Mexico, there are two distinct periods with a high number of events, with a first peak around March and a second peak around September to October. This second peak corresponds with the TC season, whereas the first peak corresponds with the passage of cold fronts and ETCs.

## Limitations and Directions for Future Research

We developed a complete reanalysis of extreme sea levels for the western North-Atlantic coasts for the period 1988–2015, including tides, and surges from TCs and ETCs. To achieve this, we improved upon the global framework of Muis *et al*.^25^ by explicitly modelling TC surges using a global hydrodynamic model (GTSM) forced with high-resolution wind and pressure fields from a parametric TC model. The validation demonstrates that the model framework can accurately reproduce large-scale spatial patterns of sea level extremes. There are a number of limitations of the model framework. First, the parametric TC model provides an idealized representation of the structure of TCs, and as a result, wind fields of TCs with an asymmetric structure or wind fields of TCs that are transitioning to an ETC are not fully resolved. We found that reanalysis data can provide good estimations for the outer TC regions, which are generally underestimated by parametric TC models. As such, there is an improvement in performance for TCs when combining the EBTRCK and ERA-Interim simulations. Further improvement of the framework could be achieved by blending of winds from the parametric model with wind from reanalysis data^62^, rather than combining the TC and ETC surge simulations from EBTRCK and ERA-Interim. In an operational context we could make use of high-resolution climate forecasts, which do capture TC intensities^66^. Second, the application of GTSM is limited in some areas. This included areas with a complex bathymetry, like estuaries and semi-enclosed bays. Accurate modelling of extremes in such areas would require a higher model resolution and the inclusion of other physics, such as river inflow and density-driven currents. Application is also limited in regions where tide-surge interaction is important, like the coast of South Carolina and Louisiana^29^. However, the contribution of nonlinear tide-surge interaction is often moderate in case of the high storm surges. The contribution of waves is also ignored, although waves can contribute greatly to extremes^28^, especially in many of the Caribbean Islands that are characterized by steep offshore slopes^67,68^. Marsooli and Ning^29^ show that the maximum wave setup is relatively large in most coastal regions (tens of cm), but that it often does not coincide with the peak of the surge and tide. During extreme events they found the contribution of the wave setup to be generally below 17%. assess the risk of coastal flooding at the local-scale^69,70^.

The dataset does not include information to reliably estimate the return periods of the most extreme events due to its relatively short length^63^. Various methods have been developed to generate large numbers of synthetic TCs that are consistent with the statistical properties of observed TCs e.g^71^. To date, these methods have been primarily applied at the local-scale. By coupling the current model framework with synthetic TCs, we aim to develop flood return periods along the western North-Atlantic coasts in future research. Such a dataset would further improve the understanding of the spatial variability of the probabilities of extreme sea levels, for example by assessing for which return periods TCs or ETCs are more important drivers^24,72^. Furthermore, return periods can be used to assess coastal flood risk at basin-scale including future sea-level rise projections^73,74^. Finally, as our approach is based on a global hydrodynamic model, the analysis can be extended to the global scale, enabling global scale risk estimates that include TC surges.

## Conclusions

We apply the 28-year reanalysis dataset to analyze the spatiotemporal patterns and drivers of extreme sea levels along the western North-Atlantic coasts. Along the US Northern Atlantic coasts the average contribution of tides to extreme sea level during the 10 largest events is up to 55% in some locations, whereas along the Mexican Southern Gulf coast, the average contribution of tides over the largest 10 events is generally below 25%. At mid-latitudes, ETCs dominate the 10 largest extreme events, whereas in the southern parts TCs dominate. During the TC season, there is a larger fraction of TC-driven events with return periods that exceed 10 years. During winter, there is a peak in ETC-driven events. Analyzing correlations between annual percentiles across sites shows there is some spatial coherence, which is more pronounced in the surge levels than the total water levels. We believe the developed reanalysis dataset can complement other datasets such as SURGEDAT^58,75,76^, and analyses based on tide gauge data e.g^9,11^. Other potential applications of the reanalysis dataset include the assessment of large-scale flood inundation and damages^77^. Furthermore, we believe the validation of the model framework is an important step towards applications to synthetic tracks for historical and future climates, as well as applications at the global scale.

## Supplementary information

Supplementary Figures and Tables
