# Supplementary material for: Spatiotemporal patterns of extreme sea levels along the western North-Atlantic coasts
Source: Sci Rep. 2019 Mar 4;9:3391. doi: 10.1038/s41598-019-40157-w (PMC6399338; doi:10.1038/s41598-019-40157-w)
Supplement: Supplementary file 1 — Supplementary Figures and Tables [file 41598_2019_40157_MOESM1_ESM.docx]

Supporting Information for

Spatiotemporal patterns of extreme sea levels along the western North-Atlantic coasts

Sanne Muis^1^, Ning Lin^2^, Martin Verlaan^3,4^, Hessel C. Winsemius^3^, Philip J. Ward^1^ & Jeroen C.J.H. Aerts^1^

^1^ Institute for Environmental Studies (IVM), Vrije Universiteit Amsterdam, Amsterdam, The Netherlands

^2^ Department of Civil and Environmental Engineering, Princeton University, Princeton, New Jersey, USA

^3^ Deltares, Delft, The Netherlands

^4^ TU Delft, Delft, The Netherlands


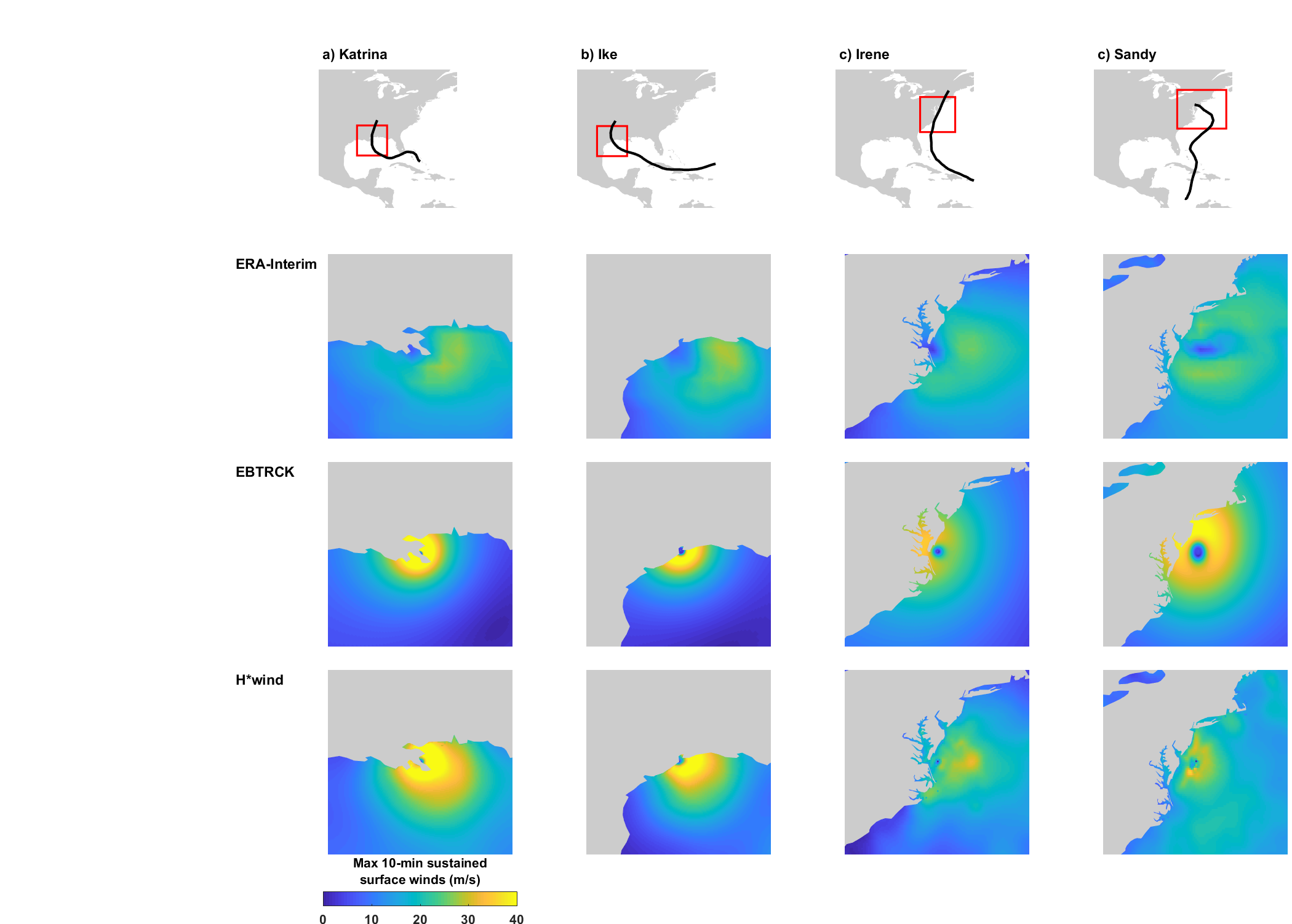


Figure S1. Evaluation of maximum 1-minute sustained surface winds (m s-1) for four major TCs. The upper, middle and lower panel show the wind intensities from ERA-Interim, EBTRCK, and H*wind for a) Hurricane Katrina, b) Hurricane Ike, c) Hurricane Irene, and d) Hurricane Sandy.


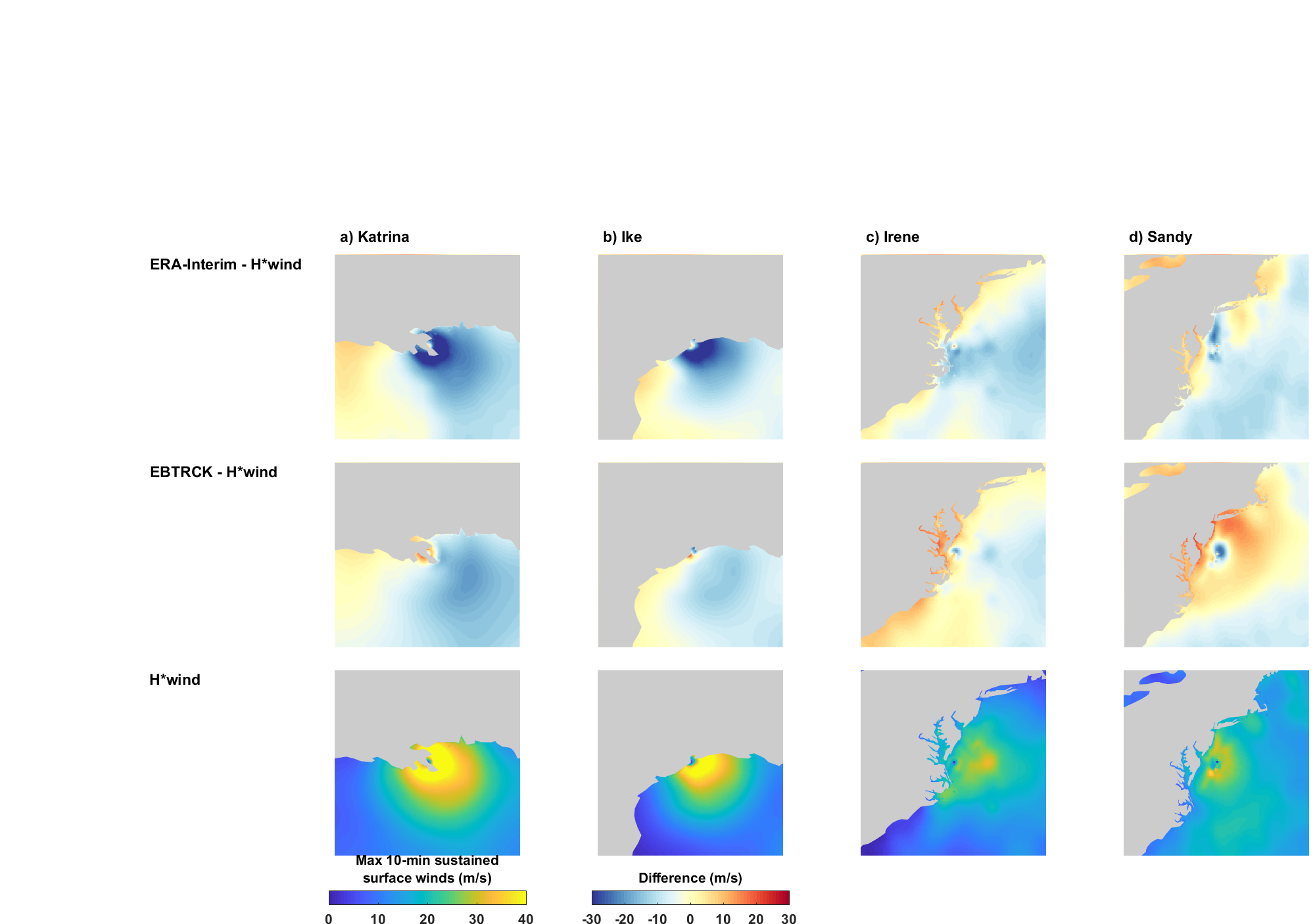


Figure S2. Evaluation of maximum 1-minute sustained surface winds (m s-1) for four major TCs. The upper, middle and lower panel show the wind intensities for ERA-Interim, EBTRCK, and H*wind for a) Hurricane Katrina, b) Hurricane Ike, c) Hurricane Irene, and d) Hurricane Sandy. The red rectangle in Figure S2 indicates the spatial domain of each panel.


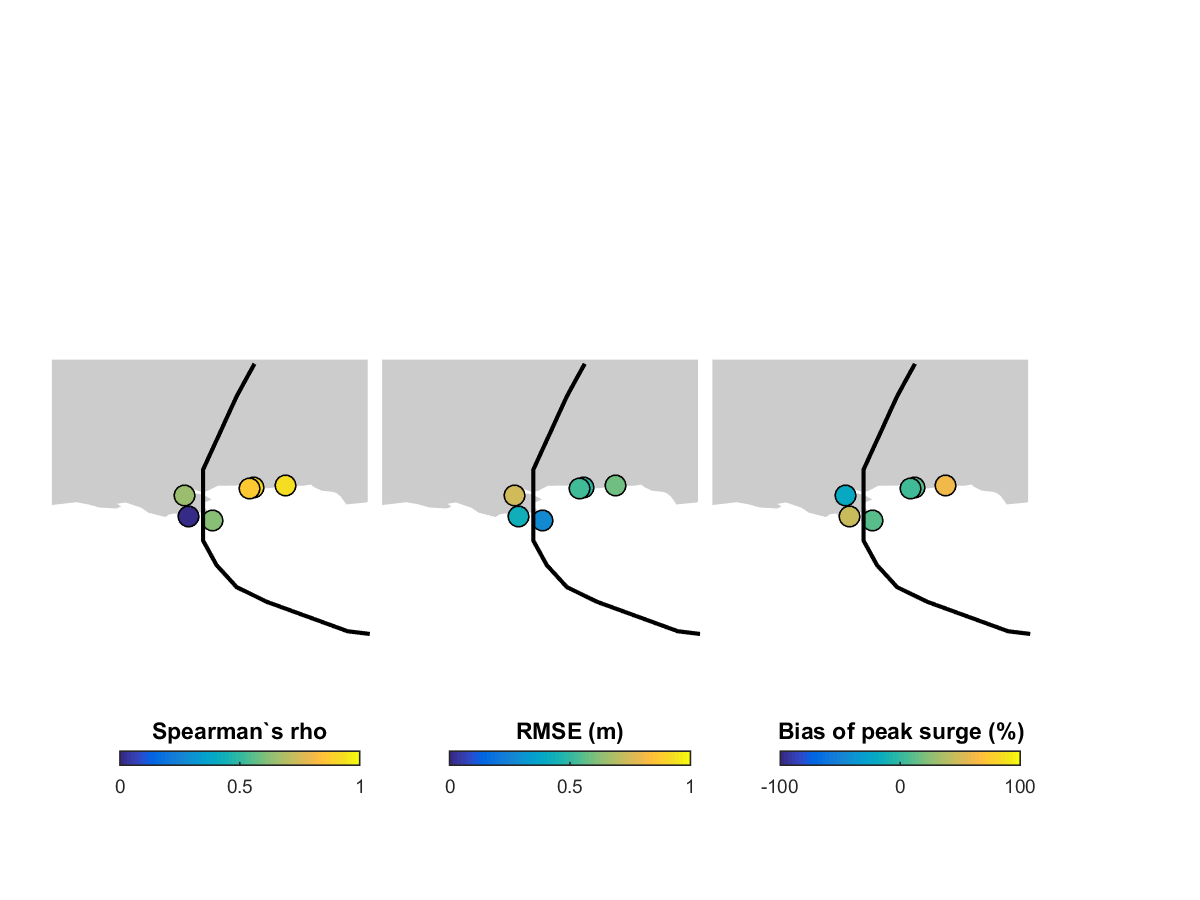


Figure S3. Model performance for Hurricane Katrina for EBTRCK expressed as the Spearman's rho, RMSE (m), and the mean bias of the peak surge (%) for available observations.


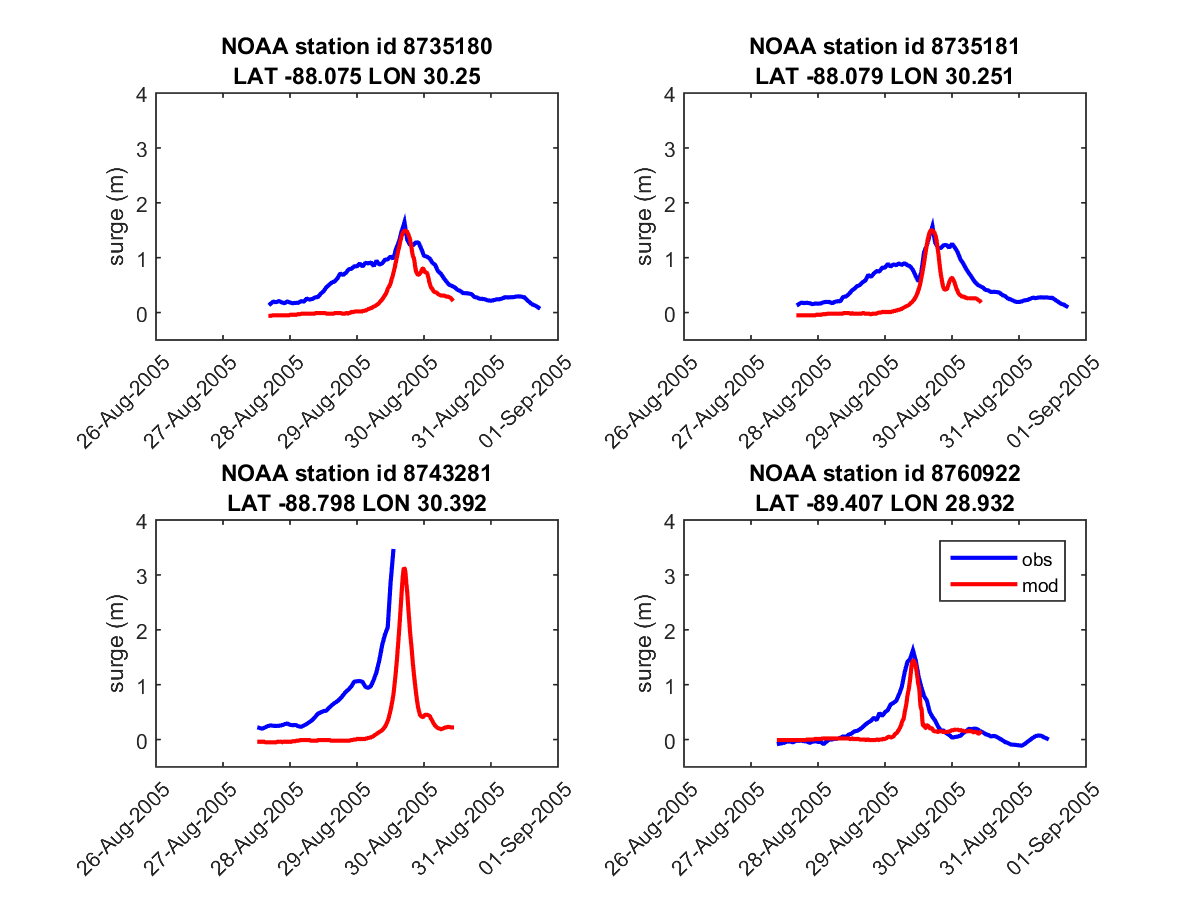


Figure S4. Time series of surge levels for EBTRCK for selected tide gauge stations during Hurricane Katrina (blue = observed; red= simulated).


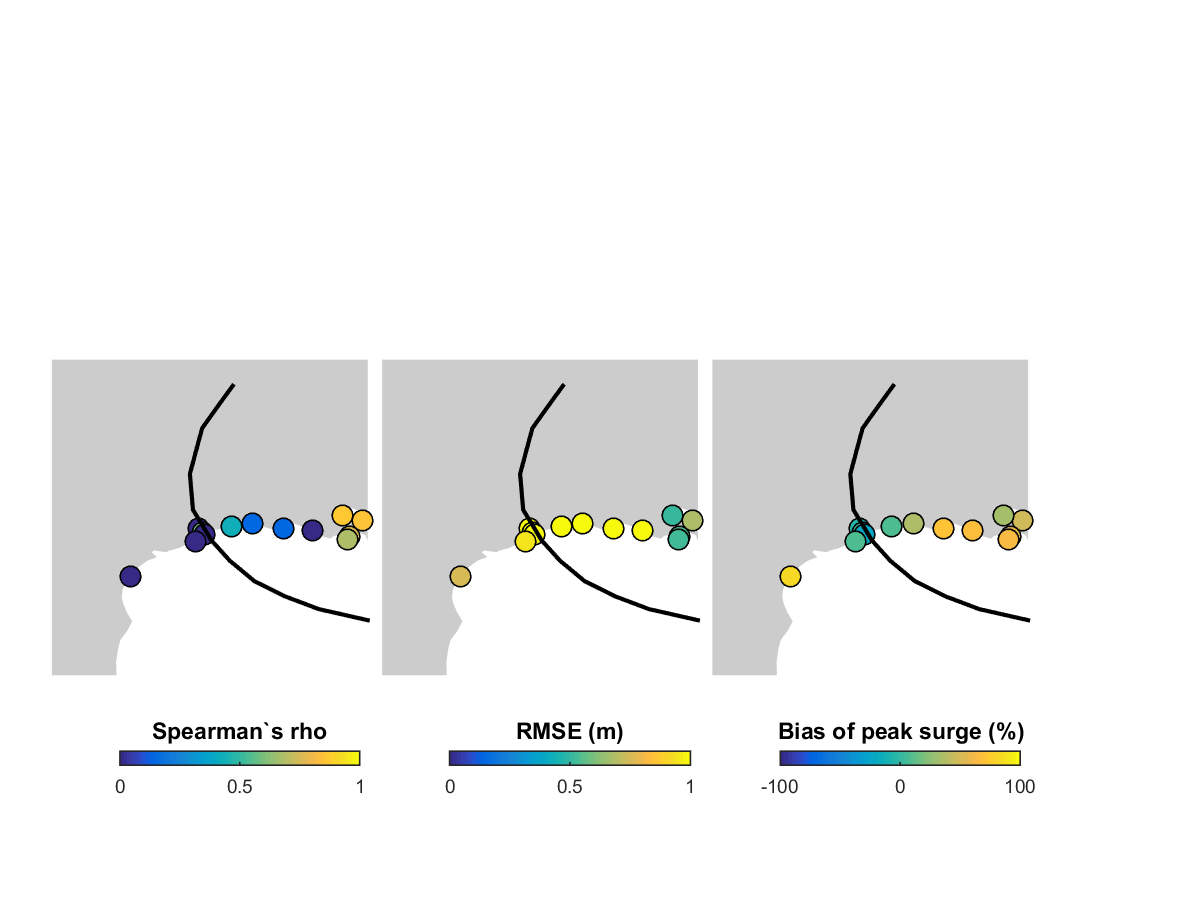


Figure S5. Model performance for Hurricane Ike for EBTRCK expressed as the Spearman's rho, RMSE (m), and the mean bias of the peak surge (%) for available observations.


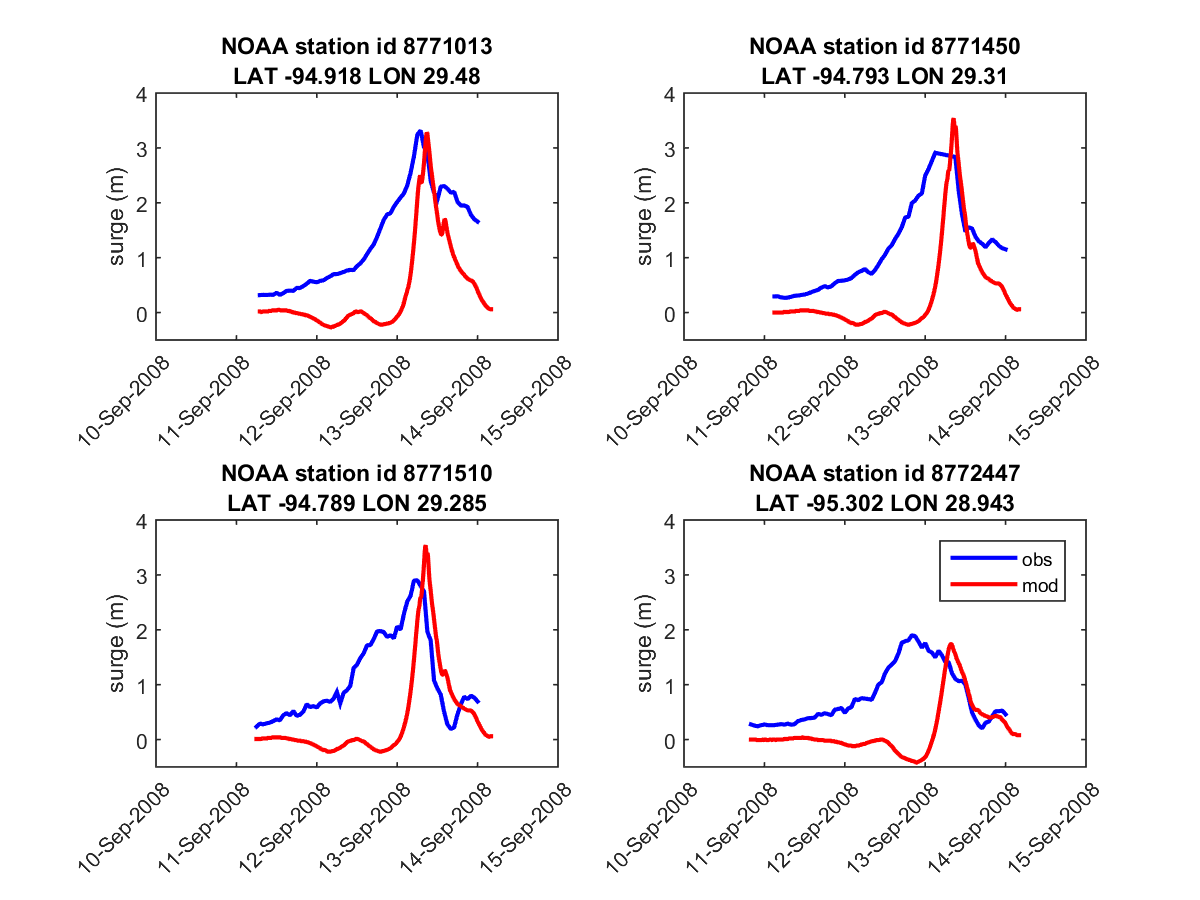


Figure S6. Time series of surge levels during Hurricane Ike for EBTRCK for selected tide gauge station (blue = observed; red= simulated).


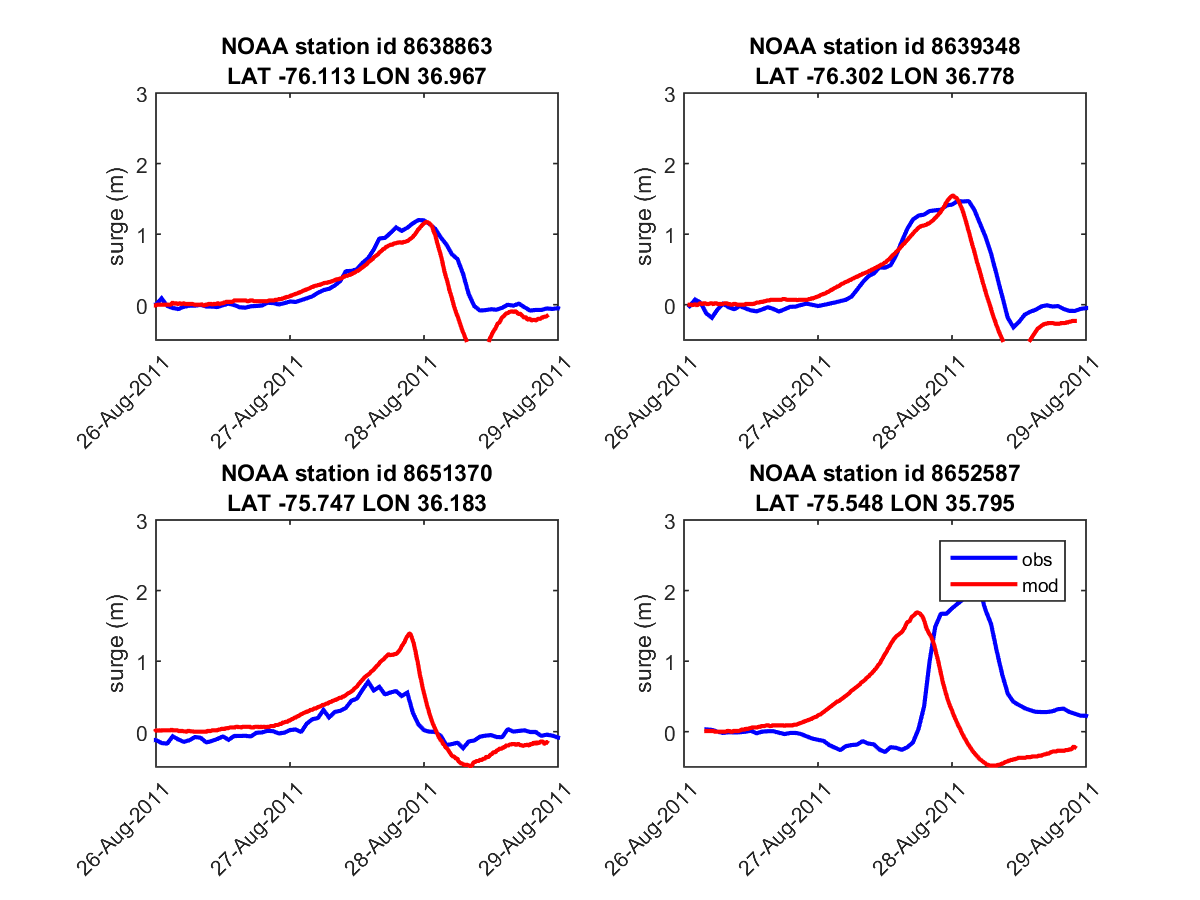


Figure S7. Time series of surge levels for EBTRCK for selected tide gauge stations during Hurricane Irene (blue = observed; red= simulated).


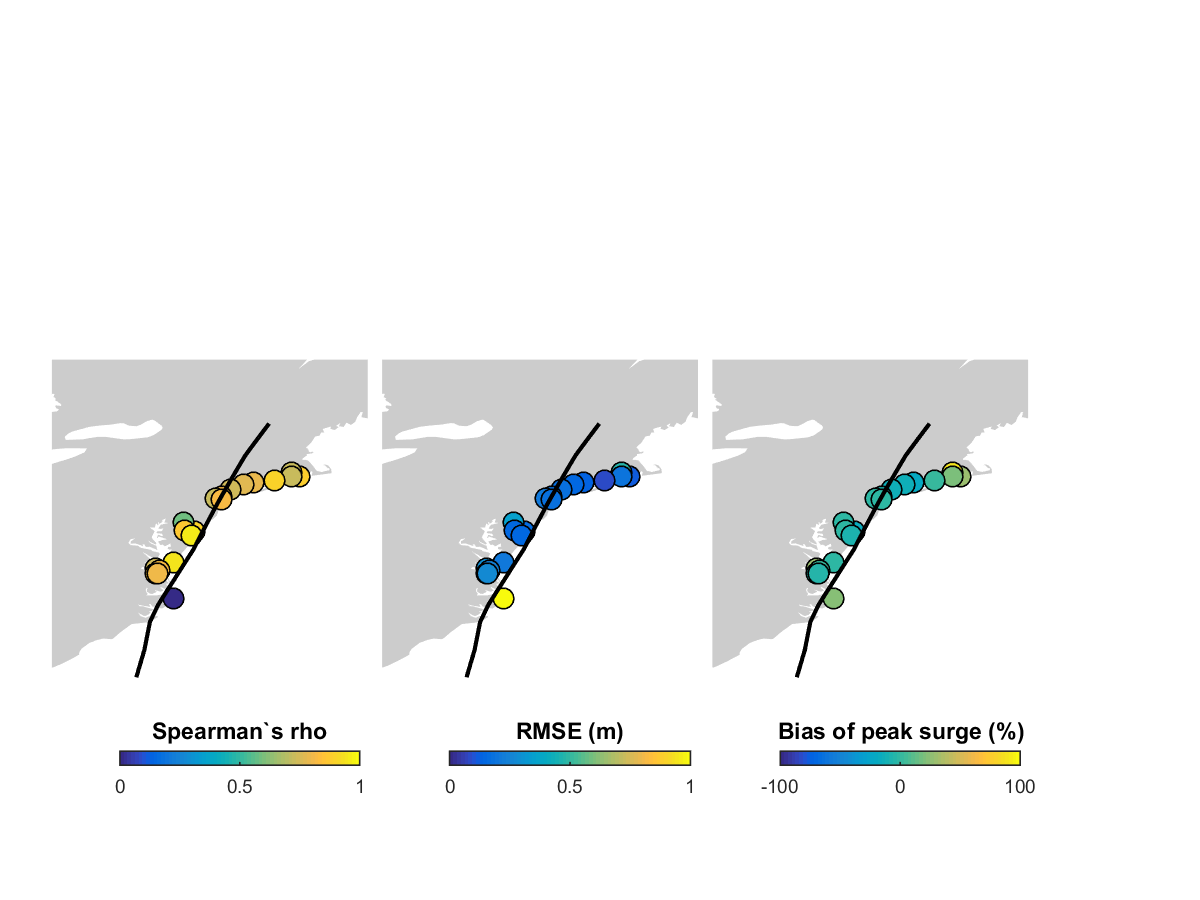


Figure S8. Model performance for Hurricane Irene expressed as the Spearman's rho, RMSE (m), and the mean bias of the peak surge (%) for available observations.


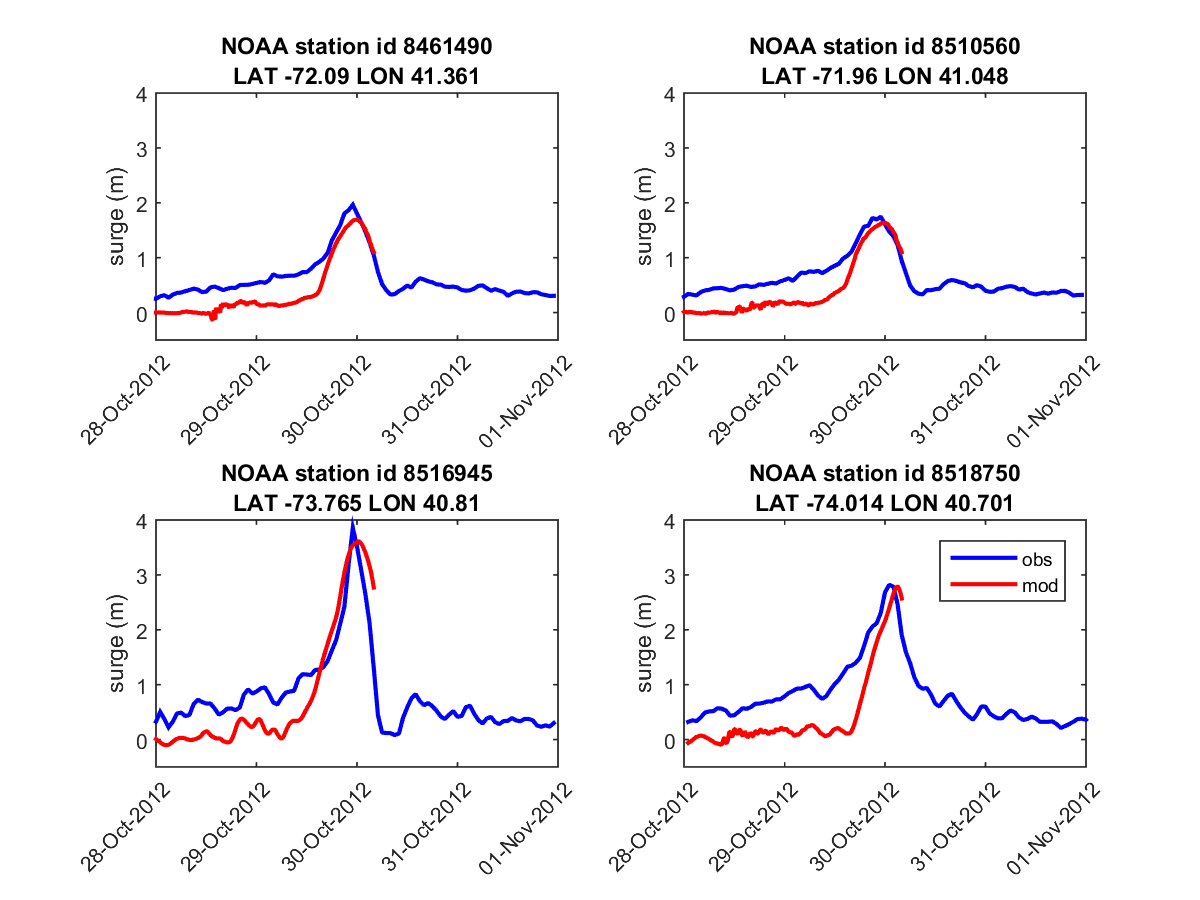


Figure S9. Time series of surge levels for EBTRCK for selected tide gauge stations during Hurricane Sandy (blue = observed; red= simulated).


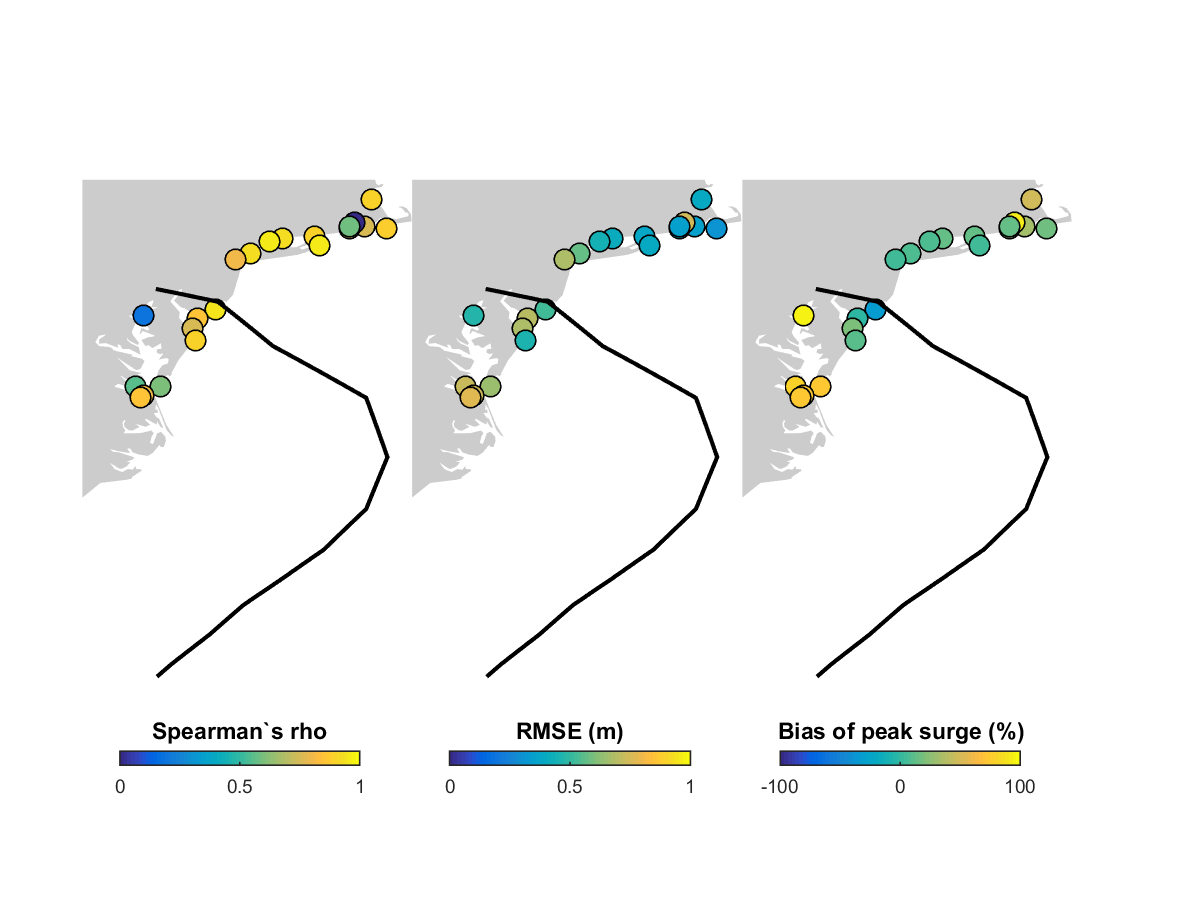


Figure S10. Model performance for Hurricane Sandy expressed as the Spearman's rho, RMSE (m), and the mean bias of the peak surge (%) for available observations.


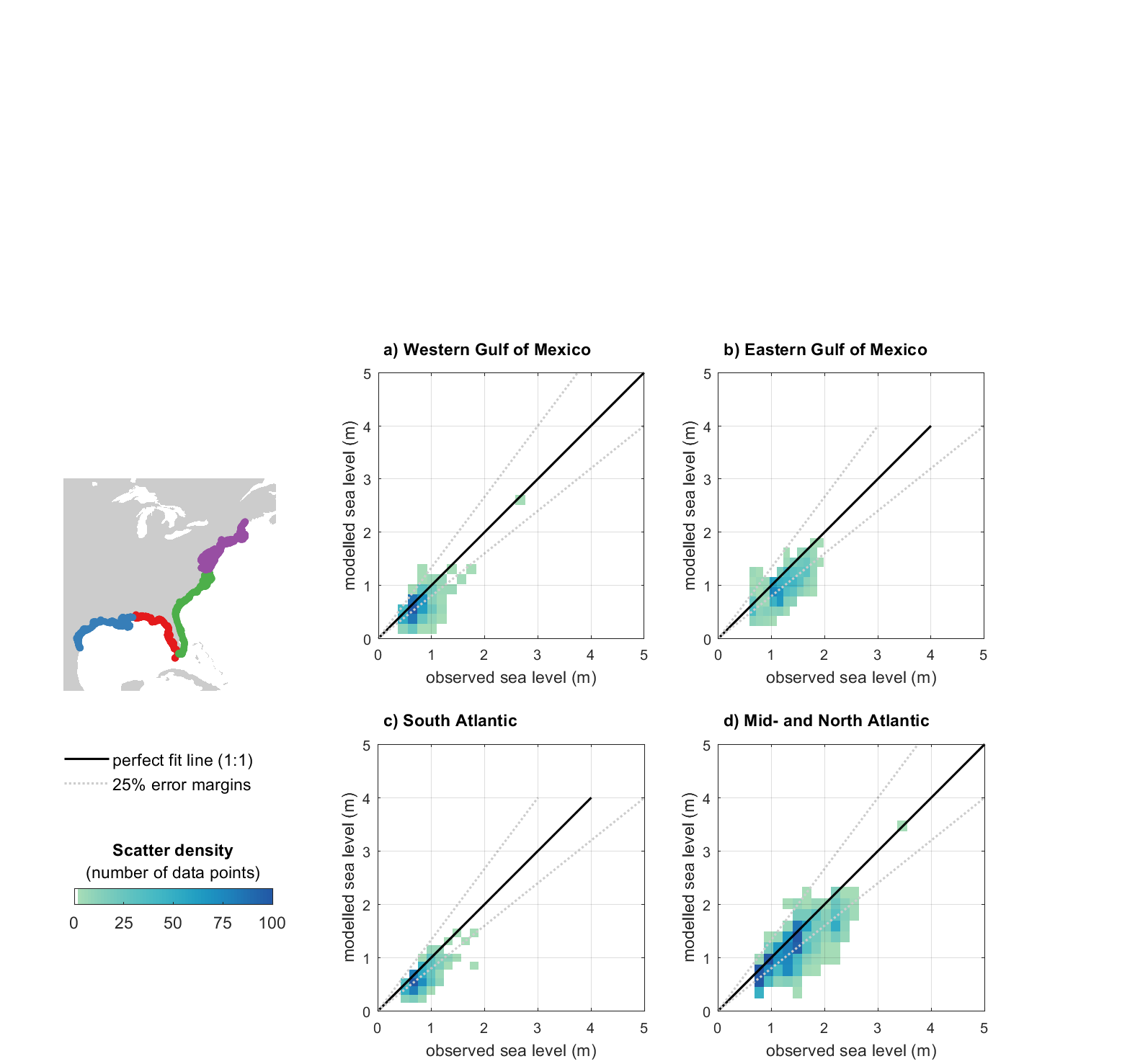


Figure S11. Scatter density plots of modeled and observed maxima for total sea levels derived from TC and ETC surge, and tides (EBTRCK, ERA-Interim and FES2012) for different regions. Results are shown for a) Western Gulf of Mexico, b) Eastern Gulf of Mexico, c) South Atlantic and d) Mid- and North Atlantic. Colors express the data density using 0.20m x 0.20m bins. The solid black line depicts the perfect fit, while the solid grey line depict the 25% error margins.

Table S1. Performance of the modeled surge and total sea levels for all Caribbean and Atlantic Coast regions, in comparison with NOAA tide gauge observations. The TC events are the simulations from the TC tracks from EBTRCK. The TC and ETC events are based on peak-over-threshold assuming an average of 3 events per year. ^.^EBTRCK and ERA-Interim were merged by taking the highest surge for each time step. S.D. denotes the standard deviation.

| **TC events** | **Tide gauge stations included^1^** | **Distance from TC track** | **Number of observations** | **Hit rate**  **(%)** | **Mean bias (m)** | **Pearson's r** |
| --- | --- | --- | --- | --- | --- | --- |
| Surge levels (EBTRCK) | 1 | <500 km | 2003 | 18 | 0.15  S.D. 0.28 | 0.53 |
|  | 2 | <500 km | 758 | 25 | 0.08  S.D. 0.21 | 0.74 |
|  | 1 | <250 km | 1146 | 33 | 0.07  S.D. 0.32 | 0.72 |
| Surge levels (EBTRKC and ERA-Interim) | 1 | <500 km | 2487 | 36 | 0.06  S.D. 0.2 3 | 0.77 |
|  | 2 | <500 km | 984 | 38 | 0.01  S.D. 0.14 | 0.88 |
|  | 1 | <250 km | 1275 | 43 | 0.01  S.D. 0.26 | 0.81 |
| **TC and ETC events** |  |  |  |  |  |  |
| Total sea levels (EBTRKC, ERA-Interim, and FES2012) | 1 | <500 km | 4957 | 59 | 0.22  S.D. 0.26 | 0.89 |
|  | 2 | <500 km | 1857 | 48 | 0.11  S.D. 0.14 | 0.84 |

*^1^ A value of 1 indicates that all tide gauge stations are included, whereas a value of 2 indicates that tide gauge stations that are located behind barrier island, in estuaries etc. are excluded.*


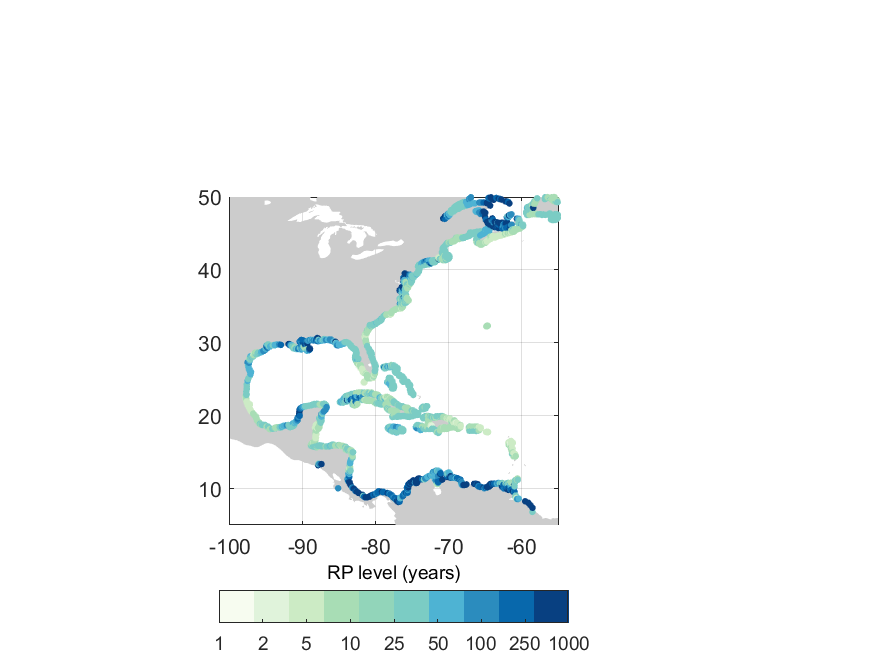


Figure S12. Map showing at which return period the GDP fit based on the surge peaks derived from EBTRCK and ERA-Interim combined exceed the GDP fit based on the surge peaks derived from ERA-Interim.
